# Age-Related Relationships between Innate Immunity and Plasma Carotenoids in an Obligate Avian Scavenger

**DOI:** 10.1371/journal.pone.0141759

**Published:** 2015-11-06

**Authors:** Isabel López-Rull, Dámaso Hornero-Méndez, Óscar Frías, Guillermo Blanco

**Affiliations:** 1 Departamento de Ecología Evolutiva, Museo Nacional de Ciencias Naturales-CSIC, Madrid, España; 2 Departamento de Biotecnología de Alimentos, Instituto de la Grasa-CSIC, Sevilla, España; CSIC-EEZA, SPAIN

## Abstract

Variation in immunity is influenced by allocation trade-offs that are expected to change between age-classes as a result of the different environmental and physiological conditions that individuals encounter over their lifetime. One such trade-off occurs with carotenoids, which must be acquired with food and are involved in a variety of physiological functions. Nonetheless, relationships between immunity and carotenoids in species where these micronutrients are scarce due to diet are poorly studied. Among birds, vultures show the lowest concentrations of plasma carotenoids due to a diet based on carrion. Here, we investigated variations in the relationships between innate immunity (hemagglutination by natural antibodies and hemolysis by complement proteins), pathogen infection and plasma carotenoids in nestling and adult griffon vultures (*Gyps fulvus*) in the wild. Nestlings showed lower hemolysis, higher total carotenoid concentration and higher pathogen infection than adults. Hemolysis was negatively related to carotenoid concentration only in nestlings. A differential carotenoid allocation to immunity due to the incomplete development of the immune system of nestlings compared with adults is suggested linked to, or regardless of, potential differences in parasite infection, which requires experimental testing. We also found that individuals with more severe pathogen infections showed lower hemagglutination than those with a lower intensity infection irrespective of their age and carotenoid level. These results are consistent with the idea that intraspecific relationships between innate immunity and carotenoids may change across ontogeny, even in species lacking carotenoid-based coloration. Thus, even low concentrations of plasma carotenoids due to a scavenger diet can be essential to the development and activation of the immune system in growing birds.

## Introduction

Immunity, defined as the capacity to fight or control parasitic or pathogenic infection, is one of the major physiological mechanisms regulating survival and a crucial determinant of fitness in animals [1]. Developing an immune response entails energetic costs as well as resource-based trade-offs and life history trade-offs [1–2]. Because optimal trade-offs can change across an individual’s lifetime as a result of variation in environmental and physiological factors, determining whether age influences the relationship of the immune system with other physiological factors can yield important insights into the mechanisms shaping immunity, and how immune responses are related to other life-history traits.

One proximate trade-off based on the allocation of limited resources to immunity occurs with carotenoids, which, for vertebrates, must be acquired through diet [3] thus being limited by the environment and/or physiological uptake [4]. Carotenoids are large lipophilic molecules that, in addition to their role as colorants, have diverse biological functions due to their immunostimulant and antioxidant properties [5–8]. Consequently, a trade-off between color signals and several physiological functions has been assumed [9]. Carotenoids can modulate many traits and processes of the immune system by influencing gene expression, protecting vulnerable cells and tissues from reactive oxygen species generated during immune responses and notably enhancing lymphocyte proliferation and cytotoxic activity, cytokine and antibody production, cutaneous delayed type hypersensitivity response, and phagocytic cell activity (reviewed in [6]). Among vertebrates and particularly in birds, several studies have shown that supplementation with carotenoids boost resistance to parasite infection [10] and stimulate antibody production [11–12], T-cell mediated immune response [5,11,13], and levels of systemic nitric oxide, which may combat infection as an intracellular signaler and pro-oxidant [14]. Likewise, other studies found that immune activation can reduce circulating carotenoid levels in birds [11–12, 15–19]. Taken together, these results indicate that immunity is influenced by environmental and individual physiological conditions, and that mounting an immune response can divert carotenoids from the blood stream, allowing individuals in good condition to better afford the costs of immune defense.

Immunity has also been shown to vary ontogenetically, with younger individuals being more exposed and vulnerable to pathogens because they are confined to nests that typically harbor ectoparasites and other disease agents [20] and because their immune system is not yet fully developed [21–22]. Since the ability of carotenoids to modulate the development of the immune system begins early during embryonic and post-hatching periods [23–25], physiological trade-offs involving carotenoids may play a key role in the achievement of individual immunity at an early stage. Indeed, some experimental studies found that in young birds, supplementation with different types of carotenoids yielded a positive effect on the immune response [26–30] (but see [31]). Because the development of the avian immune system may take several days or weeks after hatching and requires prior exposure to an antigen [21], young birds must rely mainly on their innate immunity while the specificity and memory associated with the adaptive branch fully develops [32–35]. Despite such a crucial role of innate immune functions in early defense and individual survival, relationships between innate immunity and carotenoids in free-living birds have been less well investigated than those involving acquired immunity (but see [5, 7, 11] for the cellular component, and [30, 36] for the humoral component). Particularly little attention has been paid in relation to those carotenoid interactions comprising the humoral functions of immunity, such as hemagglutination by natural antibodies and hemolysis by complement proteins, which may represent a first line of defense against initial pathogen infection [37].

The idea that variation in immunity is influenced by carotenoid availability and the physiological trade-offs involving carotenoids during development raises the question of the role that carotenoids play in modulating innate immunity in species where these micronutrients are limiting resources due to diet, e.g. carnivore animals feeding on flesh poor in carotenoids [3]. Among avian scavengers, vultures (Accipitrinidae and Cathartidae families) have been shown to have low concentrations of plasma carotenoids due to their diet, which is mainly based on rotten flesh and bones of vertebrates, although carotenoids may be actively sought after as dietary supplements potentially used in color-signaling, immunity and other functions [38–39]. However, there is limited information about the identity and concentration of different circulating carotenoids in vultures [40] and, to our knowledge, no information is available for nestling vultures.

In this study we examined the potential relationships between natural variation in innate immunity and natural variation in plasma carotenoids in adults and nestlings of a wild population of griffon vultures (*Gyps fulvus*). We focused in the performance of two constitutive innate immune functions: hemagglutination by natural antibodies and hemolysis by complement proteins. Since the assessment of the ability of the immune system to mount a response against pathogens requires the consideration of the physiological state of the individuals [25], age-variation of innate immunity in relation to plasma carotenoids was examined in respect to differences in sex, nutritional condition, physiological stress, and pathogen infection. Due to different environmental and physiological conditions over lifetime, we predict age-class variations in the relationships between innate immunity (hemagglutination by natural antibodies and hemolysis by complement proteins), pathogen infection and plasma carotenoids in nestling and adult griffon vultures, with nestlings diverting more carotenoids from the blood stream to innate immunity than fully-grown individuals due to their potentially higher exposure and vulnerability to pathogens during the ontogeny of the immune system.

## Material and Methods

### Ethics statement

Our study followed ethical guidelines proposed for the Spanish Royal Decree 1205/2005 on the protection of animals used in experiments and scientific research. The study was carried out in accordance with permits from the Spanish Bird Ringing Centre (Permit Number: 530115), and the regional government of Castilla y Léon (Expte: EP/CyL/282/2013) which approved all sampling procedures as part of obtaining the field permit. The Spanish Ministry of Economy and Competitiveness (Projects: CGL2009-12753-C02-01/BOS and CGL2010-15726) approved all sampling procedures and financed the study. Vultures were captured and handled by authorized personnel. Handling time was minimized to reduce stress and no vulture became injured during capture/sampling. After manipulation vultures were released in good state where captured. Griffon vulture is not considered as endangered species in Spain.

### Study area and species

The study was conducted in the distribution range of the griffon vulture in the Central Mountains and associated canyons of the Castilian Highlands, in Ávila and Segovia provinces, central Spain. The area includes a complex of cliffs and canyons where an increasing population of griffon vultures feeds and breeds in large numbers [41–42].

The griffon vulture is a heavy (~8–10 kg), social obligate scavenger aggregating at carcasses, breeding and roosting sites in hilly areas throughout the Palearctic region. Breeding griffon vultures are year-round residents in the study area; laying begins in late December and the young fledge from June-August [41]. Females lay one egg per clutch and both sexes are responsible for incubation and feeding of the nestling until independence >100 days after hatching. In the study area, griffon vultures and other scavengers are highly dependent on livestock carrion found at carcass dumps provided by stabled livestock operations, mostly of swine and poultry [40]. Carotenoid concentrations can vary among tissues in several livestock herbivores [43] and thus scavengers should ingest variable, but always low, levels of these pigments depending on the tissue and the degree of carrion autolysis.

Adult birds are sexually monochromatic, showing a brown plumage with black remiges and rectrices. They have a nearly bald head and neck covered with white down, the base of the neck is surrounded by a ruff of white feathers, the exposed skin on the head and legs is grey and light brown and the iris color varies from dark to light brown or grey. Therefore, there is no evidence that griffon vultures allocate carotenoids to the integument. On this basis we can reasonably assume that griffon vultures do not trade-off allocation of carotenoids between body maintenance and health signaling or ornamentation.

### Fieldwork procedures

During the breeding season of 2013, a sample of vulture nests was intensively monitored as part of long-term population monitoring program [42, 44]. All observations were made by telescope at distances that avoided disturbance of the birds in the colony. When nestlings were 50–70 days old, nests were accessed with climbing gear and nestlings (*n* = 29) were sampled. Nestling sampling was done between May 15^th^ and June 24^th^. In addition, once the breeding season ended, fully-grown vultures (*n* = 54) were captured using a large cage (5x8x2 m) placed near the breeding colony and baited with livestock carcasses. Adult captures were done between October 10^th^ and December 4^th^. Both nestlings and fully-grown vultures were banded and measured for wing (±1 mm), tail (±1 mm) and tarsus length (± 0.1 mm) with rules and digital calipers, respectively, and weighed (±1g) with balances. Wing length was used as a proxy of nestling age, because this measure increase linearly over the course of development [45] and it is relatively unaffected by environmental conditions in raptors [46–47]. Fully-grown vultures were aged as morphologically adults (*n* = 47) or subadults (*n* = 7) according to general body color, bill color and especially attending to the color, length and shape of ruff feathers [45, 48]; no subadults younger than two years of age were captured. Hereafter, we will refer to all full-grown vultures as adults. A blood sample (3–5 ml) was taken from the brachial vein of nestlings and adults, transferred to vials containing heparin and kept chilled. On the day of collection, blood samples were centrifuged at 13 000 g for 10 min to obtain plasma, which was frozen at -20°C until analysis. A drop of blood was used for sexing the individuals through molecular procedures [49].

### Nutritional condition, physiological stress and pathogen infection

Nutritional condition of nestlings was quantified as body mass relative to structural body size, by calculating the scaled mass index following Peig and Green [50]. This index adjusts the mass of all individuals to the mass they would have if they had the same body size, using the equation of the linear regression of log_10_ body mass on log_10_ tarsus length, estimated by type-2 (standardized major axis; SMA) regression (three nestlings were excluded because of missing mass or tarsus data; *r* = 0.57, lower CL = 1.15, upper CL = 4.75, *α* = 0.05). Because in adults body mass and tarsus length were not correlated (F_1, 52_ = 0.003, *r* = 0.01, *p* = 0.95), body mass was used as an indicator of nutritional condition (other morphological traits such as head size and wing length were also not significantly correlated with body mass in adults, both *p*>0.05).

To evaluate the physiological stress of nestlings and adults, we recorded the number of fault bars, i.e. conspicuous feather malformations consisting of translucent bands of frayed or missing vanes of feather keratin generally running perpendicular to the rachis, in the primary rectrices. Fault bars have been suggested to reflect stressful environmental conditions during feather growth, especially those related to nutritional deficiencies [51–52]. We therefore inspected all rectrices and recorded the presence of fault bars as a proxy of stressful conditions potentially impairing growth and development in nestlings (four nestlings were excluded because fault bars were not recorded). Fault bars were used as a proxy of chronic or long-term stressful environmental conditions in adults, as tail feathers require long molting periods, even taking several years to be fully renovated. The number of fault bars was controlled for tail length.

Pathogen infection was estimated by inspecting the oral cavity of nestlings and adults and recording the presence/absence and number of lesions caused by *Candida*-like yeast. These lesions appear as prominent white-grey or yellowish nodules of variable size and circular to elliptic in form often extending into larger and ulcerated plaque-like areas, especially on the tongue and less frequently on the pharynx, palate and other parts of the oral cavity. Although this yeast and other fungi can be normal inhabitants of the upper alimentary tract of birds, the presence of lesions from these opportunistic pathogens implies an ongoing infection [53–54] and thus the activation of the immune system. Therefore, the number of lesions was used as a proxy of the intensity of pathogen infection. No ectoparasites were detected during handling.

### Determination and quantification of plasma carotenoids

Carotenoids were extracted from plasma samples as described previously [39, 55]. In brief, a plasma aliquot (100 μL) was lyophilized and the carotenoid pigments were extracted from the dry residue with 200 μL of N, N-dimethylformamide for 60 min, including sonication for 5 min every 30 min. The resulting extract was analyzed by HPLC in accordance with the procedure outlined by Mínguez-Mosquera and Hornero-Méndez [56] with some modifications [39]. The chromatographic analysis was carried out on the same day of the preparation of the extracts. All operations were carried out under dimmed light to prevent isomerization and photodegradation of carotenoids. Concentration of plasma carotenoids is expressed in μg/mL.

### Innate immunity

Immunity was assessed following the assay described by Matson et al. [57]. This assay allows the simultaneous measurement of two constitutive innate immune functions, such as a hemagglutination reaction between natural antibodies and antigens, and a hemolysis reaction of exogenous erythrocytes, which is a function of the amount of lytic complement proteins present in the sampled blood. Quantification is done by serial dilution of plasma samples and the assessment of the dilution step at which either the hemagglutination or hemolysis reaction against the same amount of rabbit blood cell suspension stopped. Briefly, the assay was carried out in 96-well round bottom assay plates (Corning Costar #3795). Twenty-five microliters of eight plasma samples were pipetted into columns 1 and 2 of the plate and 25 μl of 0.01 M phosphate buffered saline (PBS; Sigma #P3813, St Louis, MO) were added to columns 2–12. Using a multi-channel pipette, the contents of the column 2 wells were serially diluted (1:2) through column 11, resulting in dilutions ranging from 1 to 1/1024 and 25 μl in every well. The 25 μl of PBS only in column 12 served as a negative control. For the assay itself, 25 μl of a 1% rabbit blood cell suspension was added to all wells, effectively halving all plasma dilutions. Each plate was then sealed and gently vortexed for 10 s prior to incubation during which time they were floated in a 37°C water bath for 90 min. After incubation, plates were tilted at a 45° angle along their long axis for 20 min at room temperature and then scanned to record the reaction of hemagglutination by natural antibodies. Subsequently, plates were kept at room temperature for an additional 70 min and scanned for a second time to record complement-mediated maximum hemolysis. Quantification of hemagglutination and hemolysis was done by assessing the dilution stage (on a scale from 1 to 12) at which these two reactions stopped (for further details on the method, see [57–58]. In order to estimate the repeatability of hemagglutination and hemolysis quantification based on scanned pictures, 10 individuals were measured 3 times by the same observer. Both immunity measures were highly repeatable (hemagglutination: *r* = 0.89, F_9, 20_ = 24.23, *p* < 0.0001; hemolysis: *r* = 0.94, F_9, 20_ = 74.61, *p* < 0.0001; 58).

### Data analyses

Age- and sex-related differences in plasma carotenoids and physiological state (nutritional condition, physiological stress and pathogen infection) were compared using General Linear Models (GLMs) that included age-class (nestling/adult) and sex as fixed factors. In the analyses of physiological stress, tail length was included in the models as a covariate to control for the number of fault bars. Plasma carotenoids were tested independently and separating them by their nature (xanthophylls or carotenes). Yet, since carotenoids were highly inter-correlated (Table 1) we estimated total plasma carotenoids as the sum of all of them and use this variable in the following analyses.

**Table 1:** Correlation between plasma carotenoids in nestling (*n* = 29) and adult (*n* = 54) griffon vultures. Reported values are Pearson correlation coefficients. Significant correlations (P < 0.05) are shown in bold.

Nestlings (*n* = 29)	1	2	3	4	5	6	7
*trans*-Zeaxanthin (1)	1.00	0.98	0.98	0.89	0.84	0.60	0.82
*trans*-Lutein (2)		1.00	0.99	0.89	0.87	0.69	0.89
isomers of Lutein and Zeaxanthin (3)			1.00	0.92	0.88	0.69	0.88
α -Cryptoxanthin (4)				1.00	0.83	0.64	0.79
β -Cryptoxanthin (5)					1.00	0.84	0.90
Echinenone (6)						1.00	0.84
β -Carotene (7)							1.00
Adults (*n* = 54)							
*trans*-Zeaxanthin (1)	1.00	0.97	0.97	0.69	0.81	0.57	0.76
*trans*-Lutein (2)		1.00	0.98	0.67	0.73	0.59	0.83
isomers of Lutein and Zeaxanthin (3)			1.00	0.70	0.76	0.63	0.83
α -Cryptoxanthin (4)				1.00	0.57	0.65	0.61
β -Cryptoxanthin (5)					1.00	0.51	0.57
Echinenone (6)						1.00	0.74
β -Carotene (7)							1.00

The hemolysis reaction of complement proteins (hereafter hemolysis) and hemagglutination reaction by natural antibodies (hereafter hemagglutination) were not inter-correlated either in nestlings (Spearman *r* = -0.007, *p* = 0.97, *n* = 29) or in adults (Spearman *r* = -0.001, *p* = 0.99, *n* = 54). To test for potential correlations between innate immunity and total plasma carotenoids, GLMs were performed. Initial models included immunity as the dependent variable, total plasma carotenoids as covariate, sex and age-class as fixed factors and the second grade interactions between carotenoids and age-class/sex. Initial models also included as covariates physiological stress (number of fault bars) and pathogen infection (number of lesions caused by *Candida*-like yeast) in order to control for individual physiological state. Nutritional condition was calculated differently in nestlings (scaled mass index) than in adults (body mass) and therefore, their comparison was not included in these analyses. Rather, we included body mass and tarsus length as covariates. In the hemolysis reaction of complement proteins there was a significant interaction between age-class and total plasma carotenoids (see “Results”); because no relationship in one group is sufficient to get an interaction, we examined the relationship between hemolysis reaction and plasma carotenoids with GLMs performed separately for nestlings and adults. In these initial models we also introduced nutritional condition and nestling wing length as a surrogate of age (scaled mass index was not related wing length; F_1, 24_ = 2.70, *r* = 0.32, *p* = 0.11). Model selection was carried out using a backward multiple regression method in which variables were removed from the initial model when the variance explained did not significantly improve the model (α = 0.05). Residuals from all models were normally distributed. Statistical analyses were performed using SAS and STATISTICA software.

## Results

### Age and sex variation in plasma carotenoids and physiological state

We found carotenoid pigments in the plasma of all sampled individuals. The same carotenoids were found in nestlings and adults, including xanthophylls (trans-zeaxanthin, trans-lutein, cis-lutein and cis-zeaxanthin isomers, α-cryptoxanthin, β-cryptoxanthin, and echinenone) and one carotene (β-carotene) at variable but inter-correlated concentrations (Table 1). Total plasma carotenoid concentration was 41.82% higher in nestlings than in adults (Table 2). Physiological state also showed age-related differences. The prevalence of *Candida*-like yeast was higher in nestlings (72%, *n* = 29) than in adults (26%, *n* = 54, Yates corrected χ^2^ = 14.87, *p* = 0.0001). Also, the number of lesions caused by yeast in the oral cavity was almost four times higher in nestlings than adults (Table 2). The number of fault bars controlling for tail size was not different between nestlings and adults (Table 2). No sexual differences in total plasma carotenoids or in physiological state were found in nestlings (all *p* > 0.13) or adults (all *p* > 0.28).

**Table 2:** Age-related differences in physiological state and plasma carotenoids in nestling (*n* = 29) and adult (*n* = 54) griffon vultures. Significant differences between age classes from univariate GLMs are shown in bold. Statistical comparison of nutritional condition between nestlings and adults was not performed because this variable was estimated differently for each age class (scaled mass index in nestlings and body mass in adults).

	Nestlings (*n* = 29)		Adults (*n* = 54)		Comparison		
	Mean	SD	Mean	SD	*F*	*df*	*p*
Physiological state							
Nutritional condition (g)	7055.043	954.965	9078.704	614.668	-		-
Fault bars (number)	2.000	2.843	7.430	8.702	1.212	1,76	0.274
*Candida*-like lesions (number)	1.621	1.613	0.480	1.041	15.221	1,81	0.0002
Total plasma carotenoids (μg/mL)	0.555	0.516	0.320	0.228	8.329	1,81	0.005

### Relationships between immunity, physiological state and plasma carotenoids

**Fig 1:**
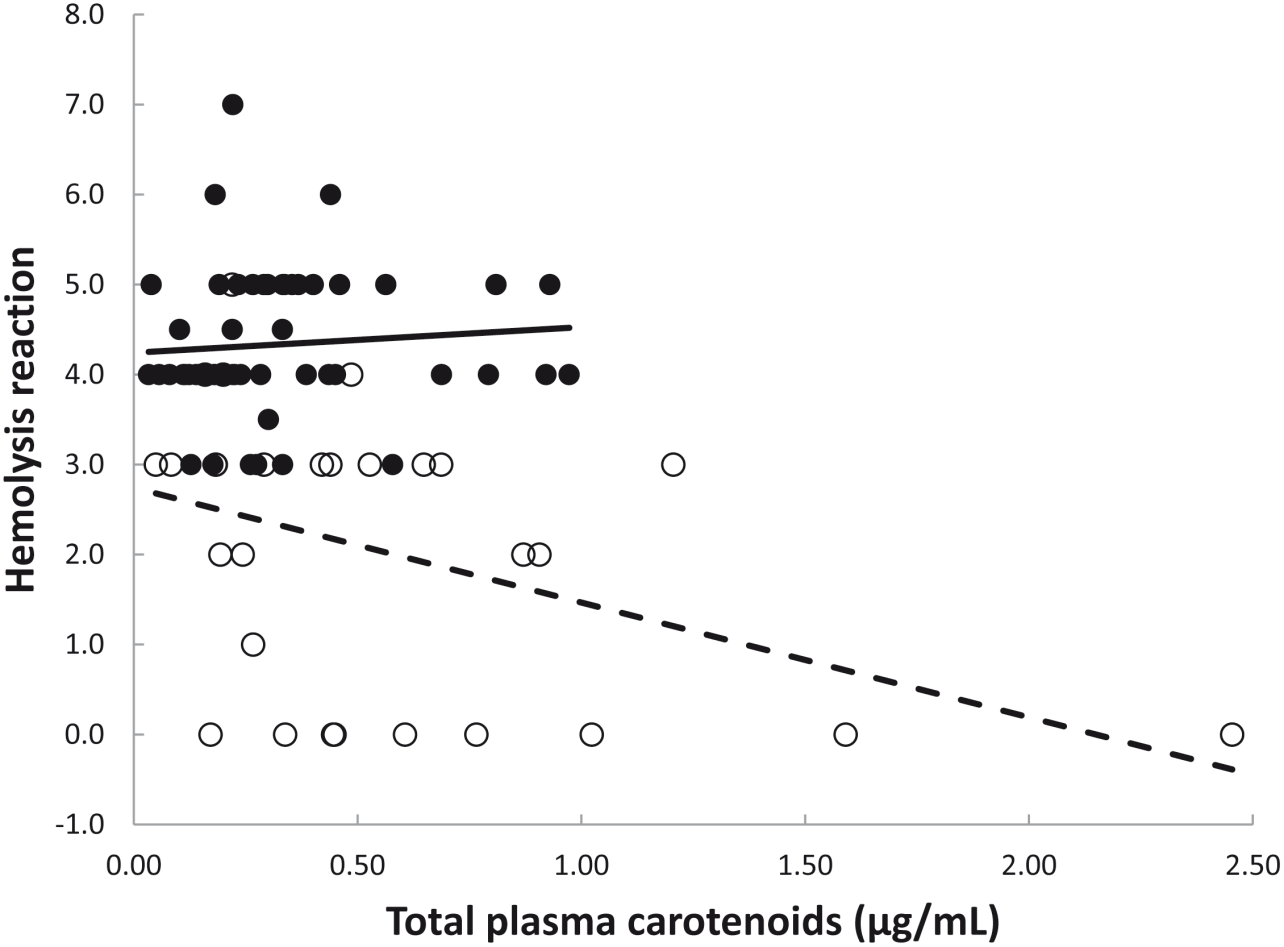
Relationship between innate immunity and plasma carotenoid concentration as a function of the hemolysis reaction of complement proteins in nestling (open circles, dotted line) and adult (solid circles, solid line) griffon vultures. Plasma carotenoids were calculated as the sum of xanthophylls (trans-zeaxanthin, trans-lutein, cis-lutein and cis-zeaxanthin isomers, α-cryptoxanthin, β-cryptoxanthin, and echinenone) and one carotene (β-carotene).

Hemolysis differed between age classes with nestlings showing around 50% lower levels than adults (nestlings: 2.03 ± 1.57, adults 4.33 ± 0.80; Table 3). Additionally, the effect of the interaction total plasma carotenoids × age-class was significant for hemolysis (Table 3; Fig 1). When nestlings and adults were tested independently we found that nestlings with lower carotenoid concentrations showed higher hemolysis levels than nestlings with higher carotenoid concentrations (F_1, 27_ = 5.71, p = 0.02), while no such a relationship was found for adults (F_1, 52_ = 0.34, p = 0.56). Hemolysis was unrelated to sex (p = 0.08), physiological stress (p = 0.27), pathogen infection (p = 0.32), body mass (p = 0.12), tarsus length (p = 0.93), nestling nutritional condition (p = 0.37) or nestling age (p = 0.60).

**Table 3:** Minimal adequate model showing the relationship between hemolysis reaction and total carotenoids (CAR), in nestling and adult griffon vultures. Significant associations are shown in bold.

	Hemolysis by complement				
	Estimate	*lower 95% CI*	*upper 95% CI*	F_1, 79_	*p*
CAR (total plasma carotenoids)	0.2851	-0.9995	1.5698	1.72	0.1941
Age class	-1.4992	-2.2736	-0.7247	14.85	**0.0002**
CAR x Age	-1.5605	-3.0654	-0.0556	4.26	**0.0423**
R^2^	0.53				

**Fig 2:**
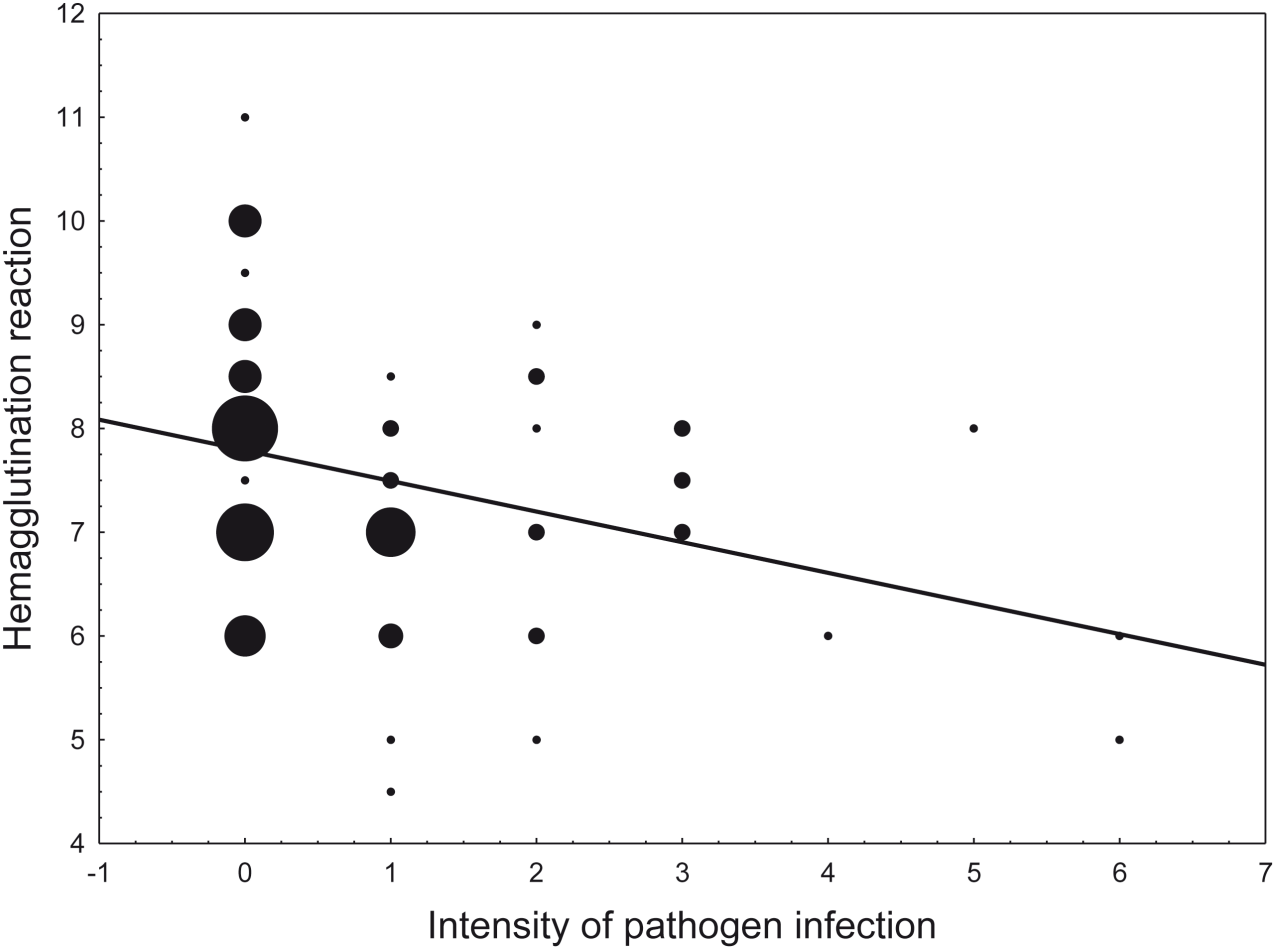
Relationship between innate immunity (as a function of the hemagglutination reaction between natural antibodies and antigens) and pathogen infection (*F*_1,81_ = 9.78, *P* = 0.002, *R* ^*2*^ = 0.10) in griffon vultures. The increasing size of multiple markers represents 1, 2, 3, 4, 5, 6, 11 and 17 cases, respectively.

The hemagglutination reaction was related to pathogen infection in both age classes (F_1, 81_ = 9.78, p = 0.002). Overall, individuals with a more severe pathogen infection showed a weaker hemagglutination reaction than those with a lower intensity infection (Fig 2). Hemagglutination was not related to age class (nestlings: 7.12 + 1.15, adults 7.75 + 1.23; p = 0.73), the interaction between age class and total plasma carotenoids (p = 0.68), total plasma carotenoids (p = 0.95), sex (p = 0.88), physiological stress (p = 0.55), body mass (p = 0.53) or tarsus length (p = 0.28).

## Discussion

Our study provides an empirical evaluation of innate immunity variability in adults and nestlings in relation to the natural variation of plasma carotenoids in a large long-lived vulture. We found age-related differences in one component of the innate immunity, plasma carotenoid concentration and pathogen infection. The main results showed that the relationship between hemolysis and carotenoids was affected different in nestlings and adults: while hemolysis levels decreased with increasing carotenoid concentration in nestlings, no such a relationship was apparent in fully-grown vultures. Furthermore, independent of age-class and carotenoid levels, individuals with a more severe pathogen infection showed weaker hemagglutination than those with a lower intensity infection. As expected by the lack of sexual dimorphism in size and morphology, and because no apparent sexual differences in habits, diet or pathogen infection in the griffon vulture exist [45, 59], the found patterns were not affected by sex.

The negative relationship between hemolysis and plasma carotenoids found in nestling griffon vultures suggests that complement activation diverted carotenoids from the plasma, as previously reported [11–12, 15–19]. Whether carotenoids were directly involved in the development of the innate immune response in nestlings or indirectly involved as a consequence of other physiological processes resulting in the activation of an immune response remains to be determined. For instance, such a relationship may be explained by a) the ability of nestlings to divert plasma carotenoids that may positively affect the magnitude of the immune response, b) a greater mobilization of plasma carotenoids due to a strong immune response, or c) a combination of the two mechanisms. In addition, because carotenoids are involved in the modulation of the immune system and the detoxification of free radicals, their mobilization in plasma might reflect a rapid re-allocation to immune tissues or cells [15] and/or short-term changes in oxidative status [18]. These two health-boosting roles may be particularly true in species lacking carotenoid coloration in which no trade-off between coloration and immunity occurs. Indeed, the low concentration of plasma carotenoids due to a diet based on carrion poor in these micronutrients suggests that carotenoid allocation to innate immunity may be essential to fighting pathogen infections. As the exposure to health threats (i.e., selection for immune development and vulnerability to pathogens) may vary between age classes, we predicted age-dependent interactions between innate immunity and circulating carotenoids. Interestingly, the negative relationship between hemolysis and plasma carotenoids was found only in nestlings, in which prevalence and intensity of pathogen infection was higher than in adults, and the concentration of circulating carotenoids was almost double. These results are consistent with the idea that different interactions between innate immunity and plasma carotenoids occur from development in the nest to adult life as a result of different environmental and physiological conditions. Also, our results may be explained by the fact that adults were raised under different infection conditions that may affect adult physiological trade-offs, raising the possibility that what we interpret as “age” may actually reflect differences in the conditions that nestlings and adult experienced during their early development. Therefore, an experimental approach is clearly needed to test these two possibilities.

It has been suggested that species that scavenge at carcasses are subject to strong parasitism and therefore have robust front line immune defenses that could potentially reduce the need for mounting relatively energetically costly lymphocyte-dependent immune responses [60]. Moreover, given the individual’s need to protect itself against pathogens, it might be expected that some types of defenses are favored over others depending on developmental stage [61–62]. It is commonly assumed that young birds rely proportionally more on their innate immunity while the specificity and memory of the adaptive branch fully develops [63–64]. However, constitutive innate immunity has also been shown to develop slowly after hatching, and is still immature just before fledging [33, 35, 57, 65–68]. Our data show that hemolysis in nestlings was significantly lower than in adults, which is in agreement with the pattern of maturation of the immune system in developing birds found in chickens (*Gallus domesticus*) [57], Leach's storm-petrels (*Oceanodroma leucorhoa*) [65], tree swallows (*Tachycineta bicolor*) [33, 68], common kestrels (*Falco tinnunculus*) [66], great tits (*Parus major*) [67] and house sparrows (*Passer domesticus*) [35]. Nonetheless, in contrast to this previous work, hemagglutination in vultures was similar between age classes. Such species-level variation in the development of constitutive immune defenses may be explained within the context of life-history theory. Recently, it has been proposed that “slow-living” species may exhibit stronger innate immunity mediated by natural antibodies, while species with ‘fast’ life histories would rely more heavily on developed complement proteins [69]. Because hemagglutination was related to pathogen infection and nestlings were more infected than adults, we propose that griffon vultures may allocate their resources to the development of natural antibodies at the expense of the slowed maturation of the immunity branch related to complement proteins. In support of this idea, we found no association between hemolysis and hemagglutination in griffon vultures, suggesting differences in the development of the innate immunity components, or a contrasting investment of each component of the innate immune systems to fight particular pathogens or variable infection intensity. Therefore, to comprehensively understand the ontogenetic interactions between innate immunity and plasma carotenoids it may be crucial to consider the likelihood of ongoing infection with the wide array of pathogens generally affecting a proportion of individuals in wild populations at variable intensities.

Overall, plasma carotenoid concentration was 41.82% higher in nestlings than in adults, and yet the carotenoid profile was similar between age classes and showed a predominance of xanthophylls (91.71% and 95.31% in nestlings and adults, respectively) in relation to carotenes (8.28% and 4.38%, respectively). As previously reported for other species of vultures [39], the identified plasma carotenoids can be considered dietary rather than metabolically derived, as they are found in fresh tissues of domestic mammal herbivores except echinenone, which is metabolically derived from β-carotene. Interestingly, the higher concentration of xanthophylls that are scarce in flesh supports the hypothesis of the consumption of dietary vegetal supplements to acquire carotenoids, either by ingesting fresh vegetation directly or indirectly via vegetal content of herbivore carcasses [39–40]. Remarkably, as compared to adults, the higher carotenoid levels in nestlings may imply a diet richer in these micronutrients supplied from vegetal matter provisioned by their parents. In addition, due to different organismal programs across ontogeny, it may be simply a consequence of the complex interactions between carotenoid availability and allocation to different physiological functions other than immunity. Both possibilities require further research.

In summary, we found that nestlings showed a weaker hemolysis reaction, higher total carotenoid concentration and more intense pathogen infection than adults. Independent of age-class and carotenoid levels, we found that individuals with more severe pathogen infection showed less immunity than those with a lower intensity infection. Taken together, these results suggest a differential carotenoid allocation to immunity due to the ongoing development of the immune system in nestlings as compared to fully-grown individuals, yet an experimental approach is needed to confirm such a possibility. These ontogenetic variations in the interactions between immunity and carotenoids can be further linked to, or may be regardless of, differential infection and its health consequences from nestling to adult life. The idea that intraspecific interactions between innate immunity and carotenoids change across ontogeny, even in species lacking carotenoid-based coloration, is supported by our data. Thus, even at low concentrations due to a scavenger diet, plasma carotenoids may play an essential role in the development and activation of the immune system in growing birds.

## Supporting Information

S1 Text DATA_Age-related relationships between innate immunity and plasma carotenoids in an obligate avian scavenger. (XLSX)
